# Machine Learning–Based Prediction of Functional Disability: a Cohort Study of Japanese Older Adults in 2013–2019

**DOI:** 10.1007/s11606-023-08215-2

**Published:** 2023-05-01

**Authors:** Yongjian Lu, Koryu Sato, Masato Nagai, Hirokazu Miyatake, Katsunori Kondo, Naoki Kondo

**Affiliations:** 1 Tokyo, Japan; 2 Department of Social Epidemiology, Graduate School of Medicine and School of Public Health, Kyoto University, Kyoto, Japan; 3 Department of Hygiene and Public Health, Osaka Medical and Pharmaceutical University, Osaka, Japan; 4 Mitsubishi Research Institute, Inc., Tokyo, Japan; 5 Department of Social Preventive Medical Sciences, Center for Preventive Medical Sciences, Chiba University, Chiba, Japan; 6 Department of Gerontological Evaluation, Center for Gerontology and Social Science, Research Institute, National Center for Geriatrics and Gerontology, Aichi, Japan

**Keywords:** machine learning, prediction model, functional disability, Japanese older adults

## Abstract

**Background:**

It is important to identify older adults at high risk of functional disability and to take preventive measures for them at an early stage. To our knowledge, there are no studies that predict functional disability among community-dwelling older adults using machine learning algorithms.

**Objective:**

To construct a model that can predict functional disability over 5 years using basic machine learning algorithms.

**Design:**

A cohort study with a mean follow-up of 5.4 years.

**Participants:**

We used data from the Japan Gerontological Evaluation Study, which involved 73,262 people aged  ≥ 65 years who were not certified as requiring long-term care. The baseline survey was conducted in 2013 in 19 municipalities.

**Main Measures:**

We defined the onset of functional disability as the new certification of needing long-term care that was ascertained by linking participants to public registries of long-term care insurance. All 183 candidate predictors were measured by self-report questionnaires.

**Key Results:**

During the study period, 16,361 (22.3%) participants experienced the onset of functional disability. Among machine learning–based models, ridge regression (*C* statistic = 0.818) and gradient boosting (0.817) effectively predicted functional disability. In both models, we identified age, self-rated health, variables related to falls and posture stabilization, and diagnoses of Parkinson’s disease and dementia as important features. Additionally, the ridge regression model identified the household characteristics such as the number of members, income, and receiving public assistance as important predictors, while the gradient boosting model selected moderate physical activity and driving. Based on the ridge regression model, we developed a simplified risk score for functional disability, and it also indicated good performance at the cut-off of 6/7 points.

**Conclusions:**

Machine learning–based models showed effective performance prediction over 5 years. Our findings suggest that measuring and adding the variables identified as important features can improve the prediction of functional disability.

**Supplementary Information:**

The online version contains supplementary material available at 10.1007/s11606-023-08215-2.

## INTRODUCTION

The world’s population of individuals aged  > 60 years will almost double between 2015 and 2050.^1^ As the population ages, functional disability becomes more prevalent. In the USA, 41.7% of people aged  ≥ 65 years reported having one or more disabilities.^2^ Functional disability is associated with adverse outcomes such as decreased quality of life and increased risks of hospitalization and mortality.^3^ However, functional declines in the aging process are dynamic and reversible. A meta-analysis reported that 13.7% of older adults improved their frailty status during the mean follow-up of 3.9 years.^4^ Therefore, it is important to identify older adults at high risk of functional disability and to take preventive measures for them at an early stage.

Several attempts have been made to predict functional disability and other functional statuses in older populations. A recent review identified 43 studies that predicted the functional status of community-dwelling older adults.^5^ In Japan, the Ministry of Health, Labour and Welfare developed a basic function checklist (the Kihon Checklist [KCL] in Japanese) comprising 25 items to identify older adults at high risk of needing long-term care. Tsuji and colleagues developed a risk score comprising ten items to predict functional disability in 3 years using data from the Japan Gerontological Evaluation Study (JAGES).^6^ Despite existing literature, there are two major research gaps. First, the variables in the developed models were selected based on expert knowledge and previous literature. Researchers can handle a limited number of potential variables and may overlook essential variables. Emerging machine learning algorithms are effective in selecting variables from many candidates without relying on a priori hypotheses or assumptions and may improve the performance of prediction models. However, none of the aforementioned studies has used these methods. Second, most of the previous studies had short follow-up periods, and only four studies from European countries followed participants for over 5 years.^5^ Because preventive measures for functional disability often need time to elicit effects, a model that can predict the distant future is necessary.

To fill these research gaps, the present study constructed prediction models of functional disability from 183 candidate predictors using machine learning algorithms. We studied functionally and cognitively independent Japanese older adults and followed them to evaluate the performance of the prediction models. To the best of our knowledge, no study has predicted functional disability using machine learning algorithms over 5 years among community-dwelling older adults.

## METHODS

### Baseline Survey

We used data from the JAGES, which studies Japanese people aged  ≥ 65 years who are not certified as needing long-term care. Self-report questionnaires were mailed to 112,705 residents in 19 municipalities across nine prefectures from October to December 2013. In ten large municipalities, participants were randomly sampled, whereas in other smaller municipalities, a census of all eligible residents was conducted. Of the invited individuals, 79,291 responded, with a response rate of 70.4%. The analysis did not include 4994 respondents, whose sex and age could not be verified. All participants provided informed consent, and the study protocol was reviewed and approved by the ethics committees of Kyoto University (R3153-2) and Nihon Fukushi University (13–14).

### Functional Disability

**Figure 1:**
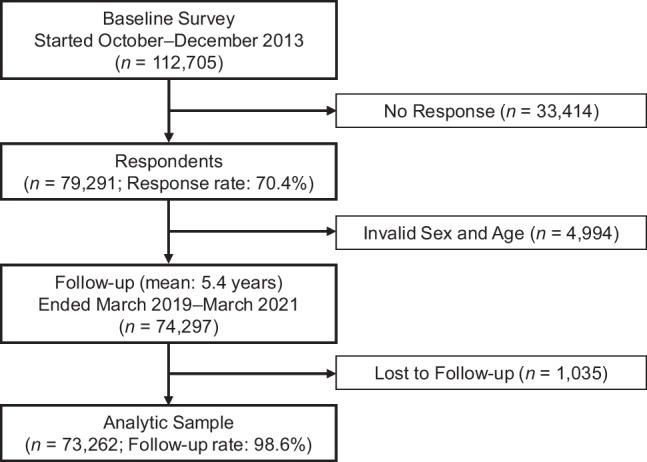
Flowchart of the analytic sample.

The onset of functional disability was defined as the new certification of needing long-term care and ascertained by linking participants to the public registries of long-term care insurance administrated by each municipality. This definition of functional disability has been widely used in previous studies.^6–10^ All Japanese citizens aged  ≥ 40 years sign up for public long-term care insurance, and they are eligible for benefits if they are determined to need care.^11^ Through a nationally standardized protocol, applicants are classified into the following eight levels of needing long-term care: not certified, support-needs levels 1–2, and care-needs levels 1–5 (larger numbers indicate severer disability; see Supplementary Table 1 for more details).^12,13^ The levels are determined according to a time estimation needed for care based on home-visit and computer-based assessments, a primary physician’s documented opinion, and a committee deliberation. In this study, those certified as one of the seven levels of needing care (except for those not certified) were considered to have functional disabilities. The follow-up period started between October and November 2013 and ended between March 2019 and March 2021 (mean follow-up, 5.4 years). Of the 74,297 eligible respondents, 73,262 participants were successfully linked to the administrative records (follow-up rate = 98.6%). Figure 1 shows a flowchart of the analytic sample.

### Candidate Predictors

We considered all variables constructed by questions that the JAGES asked all participants to be included in the prediction models. A total of 183 variables included demographic characteristics, socioeconomic status, self-reported physical and mental health, health behaviors, social capital, and community environment (see Supplementary Table 2 for the list of candidate variables). To make variables measured using different scales comparable in machine learning algorithms, they were normalized to values ranging from zero to one.

### Statistical Analysis

In general, parametric methods overweigh non-parametric methods when the relationship between an outcome and a predictor is linear, and vice versa.^14^ Thus, we examined the predictive performance of one parametric and three non-parametric machine learning algorithms: namely, ridge regression, gradient boosting, random forest, and eXtreme Gradient Boosting (XGBoost). They can be easily implemented using statistical packages and have been widely used.^14,15^ We performed logistic regression with ridge regularization to prevent overfitting by penalizing large coefficients.^16,17^ Gradient boosting^18^ and random forest^19^ are non-parametric ensemble methods that combine multiple decision trees to prevent overfitting. Whereas gradient boosting combines decision trees using boosting (iteratively correcting errors made by the previously trained tree), random forest uses bagging (bootstrap aggregating of independently developed trees). XGBoost is a new and fast algorithm of gradient boosting combining regularization.^20^

For all models, we performed a threefold cross-validation procedure; the dataset was randomly split into three groups; in the three training and validation processes, each group was always used once as test data, while the remaining groups were used as training data. Then, we repeated the same process ten times. The feature importance of selected predictors was calculated; it represents coefficients in ridge regression, while it represents relative values of reductions in the Gini index due to splits over a given predictor in other tree-based algorithms. Based on the feature importance of ridge regression, we developed a simplified risk score for functional disability to facilitate the implementation of the model (it is hard for other non-parametric models to simplify the calculation of risk scores due to non-linearity). We selected the ten most important features in the ridge regression and assigned a score of 1 to the tenth feature. Then, scores proportional to the importance were assigned to each feature (decimals were rounded off). In the dataset, 4.9% of the values were missing. To reduce the potential bias due to missing variables, we imputed them using a random forest algorithm.^21^ All analyses were performed using Python 3 (CreateSpace, Scotts Valley, CA, USA).

## RESULTS

Table 1 presents the baseline characteristics of the participants. For categorical variables, high scores indicate poor outcomes. Among the participants, 16,361 (22.3%) were newly disabled (i.e., needing long-term care) during the study period. Compared to those who remained independent, disabled people were more than 6 years older, lived with fewer household members, had lower household income, were more likely to receive public assistance, to provide self-reporting of needs for assistance in basic activities of daily living (e.g., walking, bathing, and using a toilet), to experience falls within 1 year, worry about falling, and feel bothersome, and to be diagnosed with dementia, Parkinson’s disease, and blood and immune diseases, and rated their health as poorer at baseline. In addition, disabled people were less likely to be able to climb stairs and stand up without support, engage in moderate physical activity (e.g., walking at a brisk pace, dancing, gymnastics, golf, farming, gardening, and car washing), and drive than those who remained independent. Supplementary Fig. 1 describes the distribution of certified levels of needing long-term care in the follow-up.

**Table 1:** Participants’ Characteristics

	Not needing care			Needing care		
Variables	*N*	Mean	SD	*N*	Mean	SD
Men, %	56,901	0.47	0.50	16,361	0.43	0.50
Age, year	56,901	72.53	5.39	16,361	78.71	6.21
No. of household members	53,060	2.79	1.50	14,611	2.72	1.55
Household income, 10,000JPY	46,972	234.60	152.70	12,235	207.80	144.30
Public assistance, %	54,450	0.007	0.06	15,187	0.014	0.08
Self-reported BADL	54,880	0.01	0.07	15,329	0.04	0.15
Falls within 1 year	56,088	0.12	0.26	15,897	0.22	0.34
Worried about falling, %	55,063	0.33	0.47	15,392	0.56	0.50
Self-supporting stairs climb, %	55,941	0.64	0.48	15,834	0.43	0.50
Self-supporting stand up, %	55,978	0.87	0.34	15,866	0.69	0.47
Moderate PA	52,783	0.61	0.38	14,063	0.46	0.42
Driving, %	56,802	0.59	0.49	16,304	0.36	0.48
Feeling bothersome, %	55,714	0.20	0.40	15,638	0.38	0.49
Self-rated health	55,331	0.66	0.19	15,563	0.57	0.23
Dementia, %	53,017	0.002	0.04	15,454	0.02	0.13
Parkinson’s disease, %	53,017	0.001	0.04	15,454	0.01	0.11
Blood and immune diseases, %	53,017	0.01	0.10	15,454	0.02	0.14

*SD*, standard deviation; *JPY*, Japanese Yen; *BADL*, basic activities of daily living; *PA*, physical activity

Missing values were excluded. The following categorical variables were normalized to scores ranging between 0 and 1: needs for BADL assistance, the frequency of falls within 1 year, that of moderate physical activity, and self-rated health. For the frequency of moderate physical activity and self-rated health, low scores indicate poor outcomes. Household income is equivalized by dividing by the square root of the number of household members

**Figure 2:**
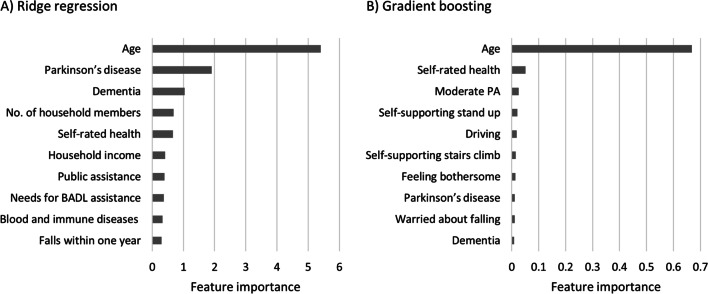
Ten important features in the prediction models. BADL, basic activities of daily living; PA, physical activity. Household income is equalized by dividing by the square root of the number of household members. Feature importance represents absolute coefficients in the ridge regression, while it represents relative values of reductions in the Gini index due to splits over a given predictor in the gradient boosting.

Table 2 compares the performance of the proposed prediction models. Among the models, ridge regression showed the best performance in predicting functional disability (*C* statistics = 0.818), whereas gradient boosting showed a similar performance (0.817). Figure 2 shows the ten most important features of the two models. In both models, we identified age, self-rated health, variables related to falls and posture stabilization, and diagnoses of Parkinson’s disease and dementia as important features. In the ridge regression, household characteristics such as the number of members, income, and receiving public assistance were also important features (Fig. 2A). In the gradient boosting model, moderate physical activity and driving also predicted functional disability (Fig. 2B).

**Table 2:** Prediction Performance for Functional Disability

Model	*C* statistic (95% CI)	*p* value^a^	Accuracy	Sensitivity	Specificity
Ridge regression	0.818 (0.812–0.824)	Reference	0.736	0.763	0.728
Gradient boosting	0.817 (0.810–0.822)	0.27	0.740	0.754	0.736
Random forest	0.803 (0.797–0.810)	< 0.001	0.728	0.746	0.722
XGBoost	0.804 (0.797–0.810)	< 0.001	0.727	0.748	0.722

*CI*, confidence interval; *XGBoost*, eXtreme Gradient Boosting

^a^*p* values for DeLong’s tests that compare *C* statistics

**Figure 3:**
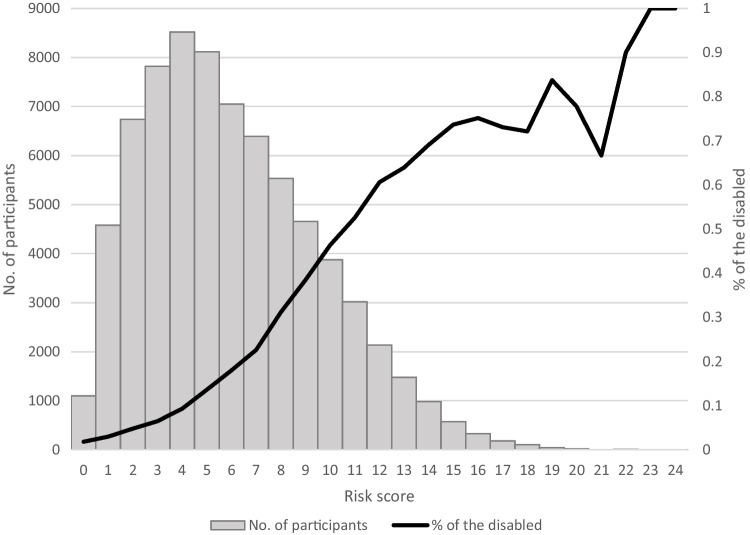
Distribution of the risk score and the percentage of the disabled.

Table 3 presents the simplified risk score for functional disability based on our ridge regression model. Figure 3 indicates the distribution of the risk score and the percentage of those who experienced the event. The continuous risk score indicated good performance (*C* statistics = 0.792). Youden index suggests that the cut-off of 6/7 points is optimal (sensitivity = 0.746, specificity = 0.699); those with a score of 7 or higher are at high risk of functional disability.

**Table 3:** Simplified Risk Score for Functional Disability Based on Ridge Regression

Variable	Importance	Score
Age, year	5.41	
65		0
66–67		1
68–69		2
70–71		3
72–73		4
74–75		5
76–77		6
78–79		7
80–81		8
82–83		9
84–85		10
86–87		11
88–89		12
90–91		13
92–93		14
94–95		15
96–97		16
98–99		17
≥ 100		18
Are you currently receiving treatment for Parkinson’s disease? (Yes)	1.91	6
Are you currently receiving treatment for dementia (e.g., Alzheimer’s disease)? (Yes)	1.04	4
Are you currently living alone? (Yes)	0.68	2
How is your current health status?	0.66	
Excellent or good		0
Fair		1
Poor		2
Is your household income less than 2 million yen? (Yes)	0.41	1
Are you currently receiving or applying for public assistance? (Yes)	0.39	1
Do you receive care or assistance for walking, bathing, or using a toilet in your daily life? (Yes)	0.37	1
Are you currently receiving treatment for blood and immune diseases? (Yes)	0.33	1
Have you had any falls over the past year? (Yes)	0.29	1

We performed several sensitivity analyses. First, we excluded participants who were certified as needing long-term care within 1 year from the baseline survey. The *C* statistics declined in all models but still showed good performance (0.809 for ridge regression and 0.807 for gradient boosting; Supplementary Table 3). Second, we tested prediction performance for the onset of severe disabilities (i.e., certified as the care-needs level 2 or severer, which requires care for basic activities of daily living), as a previous study defined.^22^ Compared to the performance for any certified needs levels, that of predicting severer conditions was lower but still good (0.805 for ridge regression and 0.804 for gradient boosting; Supplementary Table 4). Similar to our main models, both prediction models for the alternative cut-off identified age, self-rated health, and diagnoses of Parkinson’s disease and dementia as important features (Supplementary Fig. 2). In the alternative ridge regression, the use of an electric wheelchair and body mass index appeared to be important features (Supplementary Fig. 2A). In the alternative gradient boosting model, several variables related to instrumental activities of daily living (e.g., going shopping and filling out documents) were selected (Supplementary Fig. 2B). Third, we also performed a Cox proportional hazard regression. During the study period, some experienced the onset of functional disability early, others experienced late, and others were censored without the onset of functional disability. However, our main models predicted whether the participant experienced the onset of functional disability, regardless of the duration of free from it. Thus, a prediction model accounting for the time to event may better perform. Our Cox model included a penalty term using ridge regularization to prevent overfitting.^14^ The Cox model performed similarly to the logistic regression with ridge regularization and gradient boosting (0.817; Supplementary Table 5). Fourth, we confirmed whether a voting ensemble method combining the four algorithms improved performance. However, the performance improvement was slight (0.819; Supplementary Table 5). Finally, we tested the performance of the 25-item KCL, which is often used as a screening tool for those at high risk of functional disability in Japan. Although its performance was acceptable (0.716 for ridge regression and 0.717 for gradient boosting; Supplementary Table 6), our machine learning–based models performed better.

## DISCUSSION

This study constructed prediction models of functional disability using machine learning algorithms over 5 years among community-dwelling older adults. Among the models, ridge regression and gradient boosting effectively predicted functional disability. Machine learning improved prediction performance compared to models previously developed. The existing models not based on machine learning indicated median *C* statistics ranging between 0.65 and 0.76 for development models, and between 0.60 and 0.68 for validation models.^5^ While the 3-year prediction model developed by Tsuji and colleagues indicated a *C* statistic of 0.804,^6^ our model performed better with longer-term forecasts. Although the KCL (excluding five items related to depression) showed good performance in predicting functional disability in 1 year (*C* statistic = 0.83),^23^ our additional analysis suggested that its performance degrades when forecasted for more than 5 years. The simplified risk score based on our ridge regression also indicated good performance. Our findings suggest that machine learning enables us to identify those at high risk for functional disability more precisely and to take preventive measures effectively.

Several important features were identified in both models. Both models identified variables related to falls and posture stabilization as important features, namely, the frequency of falls within 1 year, worry about falls, and ability to climb stairs and stand up without support. Moreover, the models have captured the process of functional declines due to aging. People with frailty have difficulty climbing stairs and standing up on their own, and are more likely to fall.^3^ Falls and traumatic injuries increase the risk of functional disability.^24^ These four variables are also included in the KCL used in Japan’s long-term care insurance and the risk score of functional disability developed by Tsuji and colleagues.^6,23^ In line with the previous models, this study suggested that adding these measures could improve the prediction performance of functional disability. In addition, our models suggest that diagnoses of Parkinson’s disease and dementia are important predictors of functional disability as previous studies have found.^25,26^ These neurodegenerative diseases are common in the older population and result in functional impairments.^27,28^

We also found that self-rated health predicted functional disability, which was consistent with previous studies.^29–31^ Idler and Benyamini argued that there are four reasons why self-rated health can predict functional disability effectively; (1) it is more inclusive than other measures; (2) it can evaluate not only the current health status but also trajectory; (3) it affects behaviors that have an impact on future health status; and (4) it reflects about resources which one can access when health declines.^32^ Our findings suggest that self-rated health is a simple and useful measure to predict functional disability among older adults.

Furthermore, ridge regression and gradient boosting models have identified unique and important features. In the ridge regression, the household characteristics such as the number of members, income, and receiving public assistance were selected as important predictors. A previous study reported that the size of social networks, including family members, was not associated with functional disability.^33^ In contrast, the present study suggested that household size mattered, and those who experienced functional disability had a smaller household size than those who did not. Household income and the status of public assistance may reflect the socioeconomic gradient of functional disability, as previous literature showed.^34–36^

In the gradient boosting model, moderate physical activity and driving were identified as important features. Interestingly, moderate physical activity was the best predictor, although vigorous (e.g., running, swimming, cycling, tennis, exercise at the gym, and mountain climbing) and light (e.g., stretching, bowling, walking to shops or the station, and laundry) physical activities were candidate predictors in the model. Additionally, we found that in older adults, driving out of the house is a good predictor of disability. In order to prevent motor-vehicle collisions by older drivers, the Japanese National Police Agency requires drivers aged  ≥ 75 years to take a special lecture, a cognitive function test, and a driving skills test when renewing their driver’s license as well as incentivize the voluntary return of license. Given such stringent measures in Japan, driving may be a proxy variable for the retention of physical function.

There are several limitations in this study. First, objectively measured variables could not be included as candidates. Prospective studies have shown that objective measures of physical function such as gait speed, one-leg-standing time, and handgrip strength can improve the prediction of functional disability.^37,38^ Although there may be room to improve prediction performance by adding objectively measured variables, this study showed that prediction models constructed only with self-reported variables could predict functional disability with good performance. Second, this study did not provide causal models, but prediction models; thus, causality should not be inferred from our findings. There can be reverse causation and other potential biases between the identified predictors and functional disability. Readers should not interpret the results for etiology but use them to calculate the risk score of functional disability.^39^ Further studies are required to confirm causality, and to propose preventive measures for functional disability. Third, some residents did not respond to the survey, which could have caused a selection bias. We could not assess the impact of non-respondents, because we did not have this data. However, a response rate of  > 70% is comparable to or even higher than that of similar surveys involving community-dwelling older adults.^40^ Fourth, given that we aimed at predicting functional disability for individuals, clinical and biological factors were chosen as important features. However, contextual factors should also be considered for community health. Previous studies demonstrated that living in a community with active social participation and rich social cohesion was associated with the reduced onset of functional disability.^7–9,22^ Fifth, we combined all levels of needing long-term care as the outcome to predict the onset of functional disability. However, the clinical conditions of a person certified as the support-needs level 1 and a person certified as the care-needs level 5 are very different. We performed sensitivity analysis setting the care-needs level 2 as an alternative cut-off and found that the alternative models selected many of the same variables, but some were different from our primary models. We acknowledge that other prediction models may perform better to predict functional disability defined by different cut-offs and the severity of functional disability. Finally, we studied Japanese older adults, and the generalizability of our findings to other countries may be limited.

In summary, we present prediction models for functional disability that included important features selected from 183 candidate predictors using machine learning algorithms. The models showed effective performance prediction over 5 years. Our findings suggest that measuring and adding the variables identified as important features of ridge regression and gradient boosting can improve the prediction of functional disability. This study provides researchers and policymakers with valuable insights for improving the prediction of functional disabilities in community-dwelling older adults.

## Supplementary Information

Below is the link to the electronic supplementary material.

Supplementary file1 (DOCX 2559 KB)

## Data Availability

All JAGES datasets have ethical or legal restrictions for public deposition due to the inclusion of sensitive information from human participants. All enquiries are to be addressed to the data management committee via email: dataadmin.ml@jages.net.

## References

[1] World Health Organization. Ageing and health. World Health Organization. Published October 4, 2021. https://www.who.int/news-room/fact-sheets/detail/ageing-and-health.Accessed 12 Aug 2022.

[2] **Okoro CA**. Prevalence of Disabilities and Health Care Access by Disability Status and Type Among Adults — United States, 2016. MMWR Morb Mortal Wkly Rep. 2018;67. 10.15585/mmwr.mm6732a3. 10.15585/mmwr.mm6732a3 PMC6095650 30114005

[3] **Fried LP, Ferrucci L, Darer J, Williamson JD, Anderson G**. Untangling the concepts of disability, frailty, and comorbidity: implications for improved targeting and care. J Gerontol A Biol Sci Med Sci. 2004;59(3):255-263. 10.1093/gerona/59.3.m255 15031310

[4] **Kojima G, Taniguchi Y, Iliffe S, Jivraj S, Walters K**. Transitions Between Frailty States Among Community-Dwelling Older People: a Systematic Review and Meta-analysis. Ageing Res Rev. 2019;50:81-88. 10.1016/j.arr.2019.01.010. 10.1016/j.arr.2019.01.010 30659942

[5] **Van Grootven B, van Achterberg T**. Prediction Models for Functional Status in Community Dwelling Older Adults: a Systematic Review. BMC Geriatr. 2022;22(1):465. 10.1186/s12877-022-03156-7. 10.1186/s12877-022-03156-7 PMC9150308 35637447

[6] **Tsuji T, Kondo K, Kondo N, Aida J, Takagi D**. Development of a Risk Assessment Scale Predicting Incident Functional Disability Among Older People: Japan Gerontological Evaluation Study. Geriatr Gerontol Int. 2018;18(10):1433-1438. 10.1111/ggi.13503. 10.1111/ggi.13503 PMC6220974 30105800

[7] **Aida J, Kondo K, Kawachi I, et al**. Does Social Capital Affect the Incidence of Functional Disability in Older Japanese? A Prospective Population-Based Cohort Study. J Epidemiol Community Health. 2013;67(1):42-47. 10.1136/jech-2011-200307. 10.1136/jech-2011-200307 22760221

[8] **Ashida T, Kondo N, Kondo K**. Social Participation and the Onset of Functional Disability by Socioeconomic Status and Activity Type: the JAGES Cohort Study. Prev Med. 2016;89:121-128. 10.1016/j.ypmed.2016.05.006. 10.1016/j.ypmed.2016.05.006 27235600

[9] **Fujihara S, Miyaguni Y, Tsuji T, Kondo K**. Community-Level Social Participation and Functional Disability Among Older Adults: a JAGES Multilevel Longitudinal Study. Arch Gerontol Geriatr. 2022;100:104632. 10.1016/j.archger.2022.104632. 10.1016/j.archger.2022.104632 35121240

[10] **Watanabe R, Tsuji T, Ide K, et al**. Predictive Validity of the Modified Kihon Checklist for the Incidence of Functional Disability Among Older People: a 3-Year Cohort Study from the JAGES. Geriatr Gerontol Int. 2022;22(8):667-674. 10.1111/ggi.14439. 10.1111/ggi.14439 PMC9540013 35843630

[11] **Houde SC, Gautam R, Kai I**. Long-term care insurance in Japan: implications for U.S. long-term care policy. J Gerontol Nurs. 2007;33(1):7–13. 10.3928/00989134-20070101-04 17305264

[12] Ministry of Health, Labour and Welfare. Long-term care insurance system of Japan. Ministry of Health, Labour and Welfare. Published November 2016. https://www.mhlw.go.jp/english/policy/care-welfare/care-welfare-elderly/dl/ltcisj_e.pdf.Accessed 15 Sept .2022

[13] Ministry of Health, Labour and Welfare. *Older Adult Care in 2015: Toward the Establishment of Care That Supports the Dignity of Older Adults*. Ministry of Health, Labour and Welfare; 2003. https://www.mhlw.go.jp/topics/kaigo/kentou/15kourei/sankou3.html.Accessed 9 Mar 2023.

[14] **James G, Witten D, Hastie T, Tibshirani R**. *An Introduction to Statistical Learning: With Applications in R*. 2nd Edition. Springer; 2021.

[15] **Hastie T, Tibshirani R, Friedman J**. *The Elements of Statistical Learning: Data Mining, Inference, and Prediction*. 2nd edition. Springer; 2016.

[16] **Hoerl AE, Kennard RW**. Ridge Regression: Biased Estimation for Nonorthogonal Problems. Technometrics. 1970;12(1):55-67. 10.1080/00401706.1970.10488634.

[17] **Hoerl AE, Kennard RW**. Ridge Regression: Applications to Nonorthogonal Problems. Technometrics. 1970;12(1):69-82. 10.2307/1267352.

[18] **Natekin A, Knoll A**. Gradient Boosting Machines, a Tutorial. Front Neurorobotics. 2013;7:21. 10.3389/fnbot.2013.00021. 10.3389/fnbot.2013.00021 PMC3885826 24409142

[19] **Breiman L**. Random Forests. Mach Learn. 2001;45(1):5-32. 10.1023/A:1010933404324.

[20] **Chen T, Guestrin C**. XGBoost: A Scalable Tree Boosting System. In: *Proceedings of the 22nd ACM SIGKDD International Conference on Knowledge Discovery and Data Mining*. KDD ’16. Association for Computing Machinery; 2016:785–794. 10.1145/2939672.2939785.

[21] **Stekhoven DJ, Bühlmann P**. MissForest—Non-parametric Missing Value Imputation for Mixed-Type Data. Bioinformatics. 2012;28(1):112-118. 10.1093/bioinformatics/btr597. 10.1093/bioinformatics/btr597 22039212

[22] **Noguchi T, Kondo K, Saito M, Nakagawa-Senda H, Suzuki S**. Community Social Capital and the Onset of Functional Disability Among Older Adults in Japan: a Multilevel Longitudinal Study Using Japan Gerontological Evaluation Study (JAGES) Data. BMJ Open. 2019;9(10):e029279. 10.1136/bmjopen-2019-029279. 10.1136/bmjopen-2019-029279 PMC6797418 31597648

[23] **Tomata Y, Hozawa A, Ohmori-Matsuda K, et al**. Validation of the Kihon Checklist for predicting the risk of 1-year incident long-term care insurance certification: the Ohsaki Cohort 2006 Study. Nihon Koshu Eisei Zasshi Jpn J Public Health. 2011;58(1):3-13. 21409818

[24] **Tinetti ME, Williams CS**. The Effect of Falls and Fall Injuries on Functioning in Community-Dwelling Older Persons. J Gerontol Ser A. 1998;53A(2):M112-M119. 10.1093/gerona/53A.2.M112. 10.1093/gerona/53a.2.m112 9520917

[25] **Murray AM, Bennett DA, Mendes de Leon CF, Beckett LA, Evans DA**. A Longitudinal Study of Parkinsonism and Disability in a Community Population of Older People. J Gerontol Ser A. 2004;59(8):M864-M870. 10.1093/gerona/59.8.M864. 10.1093/gerona/59.8.m864 15345740

[26] **Greiner PA, Snowdon DA, Schmitt FA**. The Loss of Independence in Activities of Daily Living: the Role of Low Normal Cognitive Function in Elderly Nuns. Am J Public Health. 1996;86(1):62-66. 10.2105/ajph.86.1.62. 10.2105/ajph.86.1.62 PMC1380362 8561244

[27] **Armstrong MJ, Okun MS**. Diagnosis and Treatment of Parkinson Disease: a Review. JAMA. 2020;323(6):548-560. 10.1001/jama.2019.22360. 10.1001/jama.2019.22360 32044947

[28] **Sauvaget C, Yamada M, Fujiwara S, Sasaki H, Mimori Y**. Dementia as a Predictor of Functional Disability: a Four-Year Follow-up Study. Gerontology. 2002;48(4):226-233. 10.1159/000058355. 10.1159/000058355 12053112

[29] **Idler EL, Kasl SV**. Self-ratings of Health: Do They Also Predict Change in Functional Ability? J Gerontol B Psychol Sci Soc Sci. 1995;50(6):S344-353. 10.1093/geronb/50b.6.s344. 10.1093/geronb/50b.6.s344 7583813

[30] **Lee Y**. The Predictive Value of Self Assessed General, Physical, and Mental Health on Functional Decline and Mortality in Older Adults. J Epidemiol Community Health. 2000;54(2):123-129. 10.1136/jech.54.2.123. 10.1136/jech.54.2.123 PMC1731623 10715745

[31] **Takahashi S, Tanno K, Yonekura Y, et al**. Poor Self-rated Health Predicts the Incidence of Functional Disability in Elderly Community Dwellers in Japan: a Prospective Cohort Study. BMC Geriatr. 2020;20(1):328. 10.1186/s12877-020-01743-0. 10.1186/s12877-020-01743-0 PMC7487733 32894047

[32] **Idler EL, Benyamini Y**. Self-rated health and mortality: a review of twenty-seven community studies. J Health Soc Behav. 1997;38(1):21-37. 9097506

[33] **McLaughlin D, Leung J, Pachana N, Flicker L, Hankey G, Dobson A**. Social Support and Subsequent Disability: It Is Not the Size of Your Network That Counts. Age Ageing. 2012;41(5):674-677. 10.1093/ageing/afs036. 10.1093/ageing/afs036 22454132

[34] **Minkler M, Fuller-Thomson E, Guralnik JM**. Gradient of Disability across the Socioeconomic Spectrum in the United States. N Engl J Med. 2006;355(7):695-703. 10.1056/NEJMsa044316. 10.1056/NEJMsa044316 16914705

[35] **Zhong Y, Wang J, Nicholas S**. Gender, Childhood and Adult Socioeconomic Inequalities in Functional Disability Among Chinese Older Adults. Int J Equity Health. 2017;16(1):165. 10.1186/s12939-017-0662-3. 10.1186/s12939-017-0662-3 PMC5581446 28865465

[36] **Gjonça E, Tabassum F, Breeze E**. Socioeconomic Differences in Physical Disability at Older Age. J Epidemiol Community Health. 2009;63(11):928-935. 10.1136/jech.2008.082776. 10.1136/jech.2008.082776 19608557

[37] **Chen T, Honda T, Chen S, Kishimoto H, Kumagai S, Narazaki K**. Potential Utility of Physical Function Measures to Improve the Risk Prediction of Functional Disability in Community-Dwelling Older Japanese Adults: a Prospective Study. BMC Geriatr. 2021;21(1):476. 10.1186/s12877-021-02415-3. 10.1186/s12877-021-02415-3 PMC8411504 34470612

[38] **Guralnik JM, Ferrucci L, Simonsick EM, Salive ME, Wallace RB**. Lower-Extremity Function in Persons over the Age of 70 Years as a Predictor of Subsequent Disability. N Engl J Med. 1995;332(9):556-561. 10.1056/NEJM199503023320902. 10.1056/NEJM199503023320902 PMC9828188 7838189

[39] **Ramspek CL, Steyerberg EW, Riley RD, et al**. Prediction or Causality? A Scoping Review of Their Conflation Within Current Observational Research. Eur J Epidemiol. 2021;36(9):889-898. 10.1007/s10654-021-00794-w. 10.1007/s10654-021-00794-w PMC8502741 34392488

[40] **Santos-Eggimann B, Cuénoud P, Spagnoli J, Junod J**. Prevalence of Frailty in Middle-Aged and Older Community-Dwelling Europeans Living in 10 Countries. J Gerontol Ser A. 2009;64A(6):675-681. 10.1093/gerona/glp012. 10.1093/gerona/glp012 PMC2800805 19276189

