# Supplementary material for: Machine Learning–Based Prediction of Functional Disability: a Cohort Study of Japanese Older Adults in 2013–2019
Source: J Gen Intern Med. 2023 May 1;38(11):2486–93. doi: 10.1007/s11606-023-08215-2 (PMC10465410; doi:10.1007/s11606-023-08215-2)
Supplement: Supplementary file 1 — Supplementary file1 (DOCX 2559 KB) [file 11606_2023_8215_MOESM1_ESM.docx]

**Online Supplementary Material**

**Machine learning-based prediction of functional disability: A cohort study of Japanese older adults in 2013-19**

**Supplementary Table 1. Levels of needing care and condition profile**

| **Levels of needing care** | **Time needed for care** | **Condition profile** |
| --- | --- | --- |
| Independent  (not certified) | <25 min | The person can perform basic activities of daily living (BADL) such as walking and getting up by oneself and also perform instrumental activities of daily living (IADL) such as taking medicine and using the telephone. |
| Support-needs level 1 | 25-32 min | The person can perform most BADL but needs some support in IADL to prevent deterioration. |
| Support-needs level 2 | 32-50 min |  |
| Care-needs level 1 |  | The person has a lower ability to perform IADL than those in the support-needs levels and needs partial care for IADL. |
| Care-needs level 2 | 50-70 min | In addition to the condition of the care-needs level 1, the person needs partial care for BADL. |
| Care-needs level 3 | 70-90 min | The person has a significantly lower ability to perform both BADL and IADL than those in the care-needs level 2 and needs almost full care. |
| Care-needs level 4 | 90-110 min | The person has a lower functional ability than those in the care-needs level 3 and has difficulties in BADL without care. |
| Care-needs level 5 | ≥110 min | The person has a lower functional ability than those in the care-needs level 4 and cannot perform BADL without care. |

Reference: Ministry of Health, Labour and Welfare. Older Adult Care in 2015: Toward the Establishment of Care That Supports the Dignity of Older Adults. Ministry of Health, Labour and Welfare; 2003. Accessed March 9, 2023. https://www.mhlw.go.jp/topics/kaigo/kentou/15kourei/sankou3.html

**Supplementary Table 2. List of candidate variables**

| **Q1. We would like to ask you about your physical status.** |
| --- |
| 1) How is your current health status? |
| 1. Excellent |
| 2. Good |
| 3. Fair |
| 4. Poor |
| 2) Do you receive care or assistance for walking, bathing, and/or using a toilet in your daily life? |
| 1. I do not need nursing care or assistance. |
| 2. I need nursing care or assistance but do not receive it. |
| 3. I need and receive nursing care or assistance. |
| 3) Circle the number of all diseases for which you are currently receiving treatment or experiencing after-effects. |
| 1. High blood pressure |
| 2. Stroke (e.g. brain hemorrhage, cerebral infarction) |
| 3. Heart disease |
| 4. Diabetes |
| 5. Hyperlipidemia (lipid abnormality) |
| 6. Respiratory disease (e.g. pneumonia, bronchitis) |
| 7. Gastrointestinal, liver, or gallbladder disease |
| 8. Kidney or prostate gland disease |
| 9. Musculoskeletal disease (e.g. osteoporosis, arthrosis) |
| 10. Traumatic injury (e.g. fall, fracture) |
| 11. Cancer (malignant tumor) |
| 12. Blood or immune system disease |
| 13.Depression |
| 14. Dementia (e.g. Alzheimer's disease) |
| 15. Parkinson's disease |
| 16. Eye disease |
| 17. Ear disease |
| 18.Other ( ) |
| 19.None |
| 4) Have you been admitted to the hospital in the past year? |
| 1. No |
| 2. Yes |
| 5) How many natural teeth to you presently have? "Natural teeth" includes replanted teeth and capped teeth. Including wisdom teeth, there are a total of 32 permanent teeth. |
| 1. I have no natural teeth |
| 2. I have 1 to 4 natural teeth |
| 3. I have 5 to 9 natural teeth |
| 4. I have 10 to 19 natural teeth |
| 5. I have 20 or more natural teeth |
| 6) Do you wear dentures or bridges (non-removable dentures) or have dental implants? |
| 1. No |
| 2. Yes, in the upper jaw |
| 3. Yes, in the lower jaw |
| 4. Yes, in both jaws |
| 7) How is your ability to chew? |
| 1. I can chew anything I want. |
| 2. I can chew most foods with some exceptions. |
| 3. I can only eat limited foods as I cannot chew very well. |
| 4. I can hardly chew anything. |
| 5. I am on a liquid diet as I cannot chew at all. |
| 8) Have you ever had a check-up at a health center, your workplace, a medical institution, or another place? |
| 1. I had one within a year |
| 2. I had one within 2 to 3 years |
| 3. I had one more than 4 years ago |
| 4. I've never had one |
| **Q2. The following questions are about your eating, drinking, and smoking status.** |
| 1) Do you find chewing hard food more difficult compared to half a year earlier?* |
| 1. Yes |
| 2. No |
| 2) Have you ever choked on tea or soup?* |
| 1. Yes |
| 2. No |
| 3) Are you bothered by a feeling of thirst?* |
| 1. Yes |
| 2. No |
| 4) How often did you eat meat or fish over the past month? |
| 1. Twice a day or more |
| 2. Once a day |
| 3. Four to six times a week |
| 4. Two or three times a week |
| 5. Once a week |
| 6. Less than once a week |
| 7. None |
| 5) How often do you eat fruits and vegetables over the past month? |
| 1. Twice a day or more |
| 2. Once a day |
| 3. Four to six times a week |
| 4. Two or three times a week |
| 5. Once a week |
| 6. Less than once a week |
| 7. None |
| 6) Do you drink alcohol? |
| 1. Yes |
| 2. I used to drink |
| 3. No |
| 7) Do you smoke cigarettes? |
| 1. Yes |
| 2. I used to smoke |
| 3. No |
| **Q3. The following questions are about going out in daily life and your routine activities.** |
| 1) How often do you go out? (including to the field or immediate neighborhood, for shopping, to the hospital, etc.)* |
| 1. 4 or more a week |
| 2. Two or three times a week |
| 3. Once a week |
| 4. One to three times a month |
| 5. Several times a year |
| 6. Rarely |
| 2) Has the frequency of your going out decreased since last year?* |
| 1. Yes |
| 2. No |
| 3) What do you use for transport when you go out? Circle the numbers of all the answers that apply. |
| 1. On foot (don't use 2 to 13) |
| 2. Bicycle |
| 3. Motorcycle |
| 4. Car (drive myself) |
| 5. Car (someone else drives) |
| 6. Train |
| 7. Public bus |
| 8. Hospital or facility bus |
| 9. Wheelchair |
| 10. Motorized wheelchair (handcart) |
| 11. Walker or rollator |
| 12. Taxi |
| 13. Other ( ) |
| 4) Have you had any falls over the past year?* |
| 1. Many times |
| 2. Once |
| 3. None |
| 5) Are you very worried about falls?* |
| 1. Yes |
| 2. No |
| 6) Do you go up stairs without holding onto the handrail or the wall?* |
| 1. Yes |
| 2. No |
| 7) Do you get up out of chairs without holding anything?* |
| 1. Yes |
| 2. No |
| 8) How long do you walk a day on average? |
| 1. Less than 30 minutes |
| 2. 30 to 59 minutes |
| 3. 60 to 89 minutes |
| 4. 90 minutes or more |
| **Q4. The following questions are about your daily life. Circle the number of the appropriate answer.** |
| 1) Can you go out alone by train or bus?* |
| 1. Yes |
| 2. No |
| 2) Can you go shopping for daily necessities?* |
| 1. Yes |
| 2. No |
| 3) Can you cook for yourself? |
| 1. Yes |
| 2. No |
| 4) Can you pay your bills by yourself? |
| 1. Yes |
| 2. No |
| 5) Can you deposit or withdraw money from your bank / postal savings account(s) by yourself?* |
| 1. Yes |
| 2. No |
| 6) Can you fill out pension and other documents by yourself? |
| 1. Yes |
| 2. No |
| 7) Do you read newspapers? |
| 1. Yes |
| 2. No |
| 8) Do you read books or magazines? |
| 1. Yes |
| 2. No |
| 9) Are you interested in health-related articles or TV programs? |
| 1. Yes |
| 2. No |
| 10) Do you visit your friends’ homes?* |
| 1. Yes |
| 2. No |
| 11) Do you give advice to your family members or friends?* |
| 1. Yes |
| 2. No |
| 12) Can you visit people who have fallen ill? |
| 1. Yes |
| 2. No |
| 13) Do you start conversations with young people? |
| 1. Yes |
| 2. No |
| 14) Do you walk without stopping for about 15 minutes?* |
| 1. Yes |
| 2. No |
| 15) Do people around you notice your forgetfulness, for example, by telling you that you often ask the same thing?* |
| 1. Yes |
| 2. No |
| 16) Do you look up phone numbers and make phone calls by yourself?* |
| 1. Yes |
| 2. No |
| 17) Do you sometimes forget what date it is today?* |
| 1. Yes |
| 2. No |
| **Q5. The following questions are about your leisure activities.** |
| 1) Do you engage in any leisure activities at the moment? |
| 1. Yes |
| 2. No |
| **Q6. The following questions are about club and group participation.** |
| 1) How often do you attend activities for the following groups. |
| (1) Volunteer group |
| 1. Four or more times a week |
| 2. Two or three times a week |
| 3. Once a week |
| 4. One to three times a month |
| 5. A few times a year |
| 6. Never |
| (2) Sports group or club |
| 1. Four or more times a week |
| 2. Two or three times a week |
| 3. Once a week |
| 4. One to three times a month |
| 5. A few times a year |
| 6. Never |
| (3) Leisure activity group |
| 1. Four or more times a week |
| 2. Two or three times a week |
| 3. Once a week |
| 4. One to three times a month |
| 5. A few times a year |
| 6. Never |
| (4) Senior citizen club |
| 1. Four or more times a week |
| 2. Two or three times a week |
| 3. Once a week |
| 4. One to three times a month |
| 5. A few times a year |
| 6. Never |
| (5) Neighborhood association or residents’ association |
| 1. Four or more times a week |
| 2. Two or three times a week |
| 3. Once a week |
| 4. One to three times a month |
| 5. A few times a year |
| 6. Never |
| (6) Study or cultural group |
| 1. Four or more times a week |
| 2. Two or three times a week |
| 3. Once a week |
| 4. One to three times a month |
| 5. A few times a year |
| 6. Never |
| (7) Nursing care prevention or health-building activities |
| 1. Four or more times a week |
| 2. Two or three times a week |
| 3. Once a week |
| 4. One to three times a month |
| 5. A few times a year |
| 6. Never |
| (8) Activities to teach skills or pass on experiences to others |
| 1. Four or more times a week |
| 2. Two or three times a week |
| 3. Once a week |
| 4. One to three times a month |
| 5. A few times a year |
| 6. Never |
| (9) Local events (e.g. festivals, Bon festival dance) |
| 1. Four or more times a week |
| 2. Two or three times a week |
| 3. Once a week |
| 4. One to three times a month |
| 5. A few times a year |
| 6. Never |
| (10) Activities to support older people requiring protection |
| 1. Four or more times a week |
| 2. Two or three times a week |
| 3. Once a week |
| 4. One to three times a month |
| 5. A few times a year |
| 6. Never |
| (11) Activities to support older people requiring nursing care |
| 1. Four or more times a week |
| 2. Two or three times a week |
| 3. Once a week |
| 4. One to three times a month |
| 5. A few times a year |
| 6. Never |
| (12) Activities to support parents raising children |
| 1. Four or more times a week |
| 2. Two or three times a week |
| 3. Once a week |
| 4. One to three times a month |
| 5. A few times a year |
| 6. Never |
| (13) Local living arrangement improvement (beautification) activities |
| 1. Four or more times a week |
| 2. Two or three times a week |
| 3. Once a week |
| 4. One to three times a month |
| 5. A few times a year |
| 6. Never |
| (14) Another group or organization (if possible, please give the name and type of activity: ) |
| 1. Four or more times a week |
| 2. Two or three times a week |
| 3. Once a week |
| 4. One to three times a month |
| 5. A few times a year |
| 6. Never |
| **Q7. The following questions are about your relationships with your friends.** |
| 1) How often do you see your friends? |
| 1. Four or more a week |
| 2. Two or three times a week |
| 3. Once a week |
| 4. One to three times a month |
| 5. A few times a year |
| 6. Rarely |
| 2) How many friends/acquaintances have you seen over the past month? Count the same person as one, no matter how many times you have seen him/her. |
| 1. None |
| 2. 1 to 2 |
| 3. 3 to 5 |
| 4. 6 to 9 |
| 5. 10 or more |
| **Q8. The following questions are about mutual assistance with the people around you. Circle the numbers of all the answers that apply.** |
| 1) Do you have someone who listens to your concerns and complaints? |
| 1. Yes |
| 2. No |
| 2) Do you listen to someone’s concerns and complaints? |
| 1. Yes |
| 2. No |
| 3) Do you have someone who looks after you when you are sick and confined to a bed for a few days? Circle the numbers of all the answers that apply. |
| 1. Yes |
| 2. No |
| 4) Do you look after someone when he/she is sick and confined to a bed for a few days? Circle the numbers of all the answers that apply. |
| 1. Yes |
| 2. No |
| **Q9. The following questions are about the area where you live.** |
| 1) Do you think people living in your area can be trusted in general? |
| 1. Very |
| 2. Moderately |
| 3. Neutral |
| 4. Slightly |
| 5. Not at all |
| 2) Do you think people living in your area try to help others in most situations? |
| 1. Very |
| 2. Moderately |
| 3. Neutral |
| 4. Slightly |
| 5. Not at all |
| 3) How attached are you to the area you live? |
| 1. Very |
| 2. Moderately |
| 3. Neutral |
| 4. Slightly |
| 5. Not at all |
| 4) Which of the following changes have you seen in the area you live? Circle the numbers of all the answers that apply. |
| 1. Revitalization of the local economy |
| 2. Depression of the local economy |
| 3. Deterioration of security |
| 4. Increase of newcomers |
| 5. Decline of the local festival |
| 6. Increase in unemployment |
| 7. Increase in poverty |
| 8. Improvement of administrative services |
| 9. Deterioration of administrative services |
| 10. Widening income disparity |
| 11. Increasing interaction or activities among community residents |
| 12. Declining interaction or activities among community residents |
| 13. None (do not see any of the changes from 1 to 12) |
| 5) What kind of interactions do you have with people in your neighborhood? |
| 1. Mutual consultation, lending and borrowing daily commodities, cooperation in daily life |
| 2. Standing and chatting frequently |
| 3. No more than exchanging greetings |
| 4. None, not even greetings |
| 6) Are the following present within walking distance of your home (within about 1 km)? |
| (1) Locations with noticeable graffiti or undisposed garbage |
| 1. Many |
| 2. Some |
| 3. Few |
| 4. None |
| 5. I don’t know |
| (2) Parks or foot paths suitable for exercise or walking |
| 1. Many |
| 2. Some |
| 3. Few |
| 4. None |
| 5. I don’t know |
| (3) Locations difficult for walking, such as hills or steps |
| 1. Many |
| 2. Some |
| 3. Few |
| 4. None |
| 5. I don’t know |
| (4) Roads or crossroads with a great risk of traffic accidents |
| 1. Many |
| 2. Some |
| 3. Few |
| 4. None |
| 5. I don’t know |
| (5) Fascinating views or buildings |
| 1. Many |
| 2. Some |
| 3. Few |
| 4. None |
| 5. I don’t know |
| (6) Shops, facilities, or wagon retailers selling fresh foods (e.g. meat, fish, vegetables, fruits) |
| 1. Many |
| 2. Some |
| 3. Few |
| 4. None |
| 5. I don’t know |
| (7) Dangerous places when walking alone at night |
| 1. Many |
| 2. Some |
| 3. Few |
| 4. None |
| 5. I don’t know |
| (8) Houses or facilities you feel free to drop in |
| 1. Many |
| 2. Some |
| 3. Few |
| 4. None |
| 5. I don’t know |
| 7) How do you usually get fresh foods? Circle the number of all that apply. |
| 1. Go out by myself to go grocery shopping |
| 2. Family member takes me grocery shopping |
| 3. Use a pickup/drop-off service to go grocery shopping |
| 4. Ask a family member to do my grocery shopping |
| 5. Use a grocery shopping service (such as a housekeeper) |
| 6. Use a home-delivery service |
| **Q10. Circle the number of the appropriate answer in the following questions.** |
| 1) Are you basically satisfied with your life? |
| 1. Yes |
| 2. No |
| 2) Do you feel pretty worthless the way you are now? |
| 1. Yes |
| 2. No |
| 3) Have you dropped many of your activities and interests? |
| 1. Yes |
| 2. No |
| 4) Do you feel that your life is empty? |
| 1. Yes |
| 2. No |
| 5) Do you often get bored? |
| 1. Yes |
| 2. No |
| 6) Are you in good spirits most of the time? |
| 1. Yes |
| 2. No |
| 7) Are you afraid that something bad is going to happen to you? |
| 1. Yes |
| 2. No |
| 8) Do you feel happy most of the time? |
| 1. Yes |
| 2. No |
| 9) Do you often feel helpless? |
| 1. Yes |
| 2. No |
| 10) Do you prefer to stay at home, rather than going out and doing new things? |
| 1. Yes |
| 2. No |
| 11) Do you feel you have more problems with memory than most? |
| 1. Yes |
| 2. No |
| 12) Do you think it is wonderful to be alive now? |
| 1. Yes |
| 2. No |
| 13) Do you feel full of energy? |
| 1. Yes |
| 2. No |
| 14) Do you feel that your situation is hopeless? |
| 1. Yes |
| 2. No |
| 15) Do you think that most people are better off than you are? |
| 1. Yes |
| 2. No |
| 16) Do you feel there was no self-fulfillment in everyday life in the past two weeks?* |
| 1. Yes |
| 2. No |
| 17) Has something you used to enjoy very much become boring in the past two weeks?* |
| 1. Yes |
| 2. No |
| 18) Has something you used to do easily become bothersome in the past two weeks?* |
| 1. Yes |
| 2. No |
| 19) Have you thought you are not useful in the past two weeks?* |
| 1. Yes |
| 2. No |
| 20) Have you felt tired for no reason in the past two weeks?* |
| 1. Yes |
| 2. No |
| **Q11. The following questions are about your personal characteristics. Circle the number of the answer the best applies.** |
| 1) Sex |
| 1. Male |
| 2. Female |
| 2) Age |
| years old |
| 3) What is your current height and weight? (Give a rough estimate)* |
| Height: Approximately ( ) cm |
|  |
| Weight: Approximately ( ) kg |
| 4) Have you lost more than 2-3 kg over the past six months?* |
| 1. Yes |
| 2. No |
| 5) Do you feel that you are happy? Circle the number of the appropriate score. |
| 10 points Very happy |
| 9 points |
| 8 points |
| 7 points |
| 6 points |
| 5 points |
| 4 points |
| 3 points |
| 2 points |
| 1 point Not happy |
| 6) How long have you been living in your present home? |
| About years |
| 7) How many years of formal education have you had? |
| 1. Less than 6 years |
| 2. 6 to 9 years |
| 3. 10 to 12 years |
| 4. 13 years or more |
| 5. Other |
| 8) Have you experienced any of the following events over the past year? Circle the numbers of all answers that apply. |
| 1. I started a new job. |
| 2. I quit my job or retired. |
| 3. I started living with my children. |
| 4. I started living alone. |
| 5. I became better off financially. |
| 6. I became worse off financially. |
| 7. A new grandchild or great-grandchild was born |
| 8. I lost my spouse. |
| 9. A family member or close friend or relative passed away |
| 10. I acquired new friends. |
| 11. I suffered a serious illnesses. |
| 12. I started caring for sick family members. |
| 13. Other ( ) |
| 14. No major changes. |
| **Q12. The following questions are about physical movement in regular daily life. *Do not include movement for work.** |
| 1) How often do you exert yourself strenuously, to the extent of the following activities? |
| e.g. running, swimming, cycling, tennis, exercise at the gym, mountain climbing |
| 1. Four or more times a week |
| 2. Two or three times a week |
| 3. Once a week |
| 4. One to three times a month |
| 5. A few times a year |
| 6. Rarely |
| 2) How often do you exert yourself moderately, to the extent of the following activities? |
| e.g. walking (at a brisk pace), dancing, gymnastics, golf, farming, gardening, car washing |
| 1. Four or more times a week |
| 2. Two or three times a week |
| 3. Once a week |
| 4. One to three times a month |
| 5. A few times a year |
| 6. Rarely |
| 3) How often do you exert yourself lightly, to the extent of the following activities? |
| e.g. stretching (calisthenics), bowling, walking to shops or the station, laundry |
| 1. Four or more times a week |
| 2. Two or three times a week |
| 3. Once a week |
| 4. One to three times a month |
| 5. A few times a year |
| 6. Rarely |
| **Q13. The following questions are about your family.** |
| 1) What is your marital status? |
| 1. Married |
| 2. Widowed |
| 3. Divorced |
| 4. Never married |
| 5. Other |
| 2) Which of the following best describes your current financial living conditions as a whole? |
| 1. Very difficult |
| 2. Difficult |
| 3. Comfortable |
| 4. Very comfortable |
| 3) Which of the following best describes your family composition? |
| 1. I live alone |
| 2. I live with my family (including two-family homes) |
| 3. Other (e.g. institution) |
| 4) How many people are in your household, including yourself? |
| ( )　people |
| 5) What was your pretax annual household income for 2012 (including pension)? Circle the number of the answer that best applies. |
| 1. Less than 500,000 yen |
| 2. 500,000 to less than 1 million yen |
| 3. 1 million to less than 1.5 million yen |
| 4. 1.5 million to less than 2 million yen |
| 5. 2 million to less than 2.5 million yen |
| 6. 2.5 million to less than 3 million yen |
| 7. 3 million to less than 4 million yen |
| 8. 4 million to less than 5 million yen |
| 9. 5 million to less than 6 million yen |
| 10. 6 million to less than 7 million yen |
| 11. 7 million to less than 8 million yen |
| 12. 8 million to less than 9 million yen |
| 13. 9 million to less than 10 million yen |
| 14. 10 million to less than 12 million yen |
| 15. More than 12 million yen |
| 6) What is the total value of your household assets, including savings, real estate (e.g. house, land, condominium), stocks, golf membership, etc.? Circle the number that best represents the total value of your assets. |
| 1. Less than 500,000 yen |
| 2. 500,000 to less than 1 million yen |
| 3. 1 million to less than 5 million yen |
| 4. 5 million to less than 10 million yen |
| 5. 10 million to less than 50 million yen |
| 6. More than 50 million yen |
| 7) What type of residence do you live in? |
| 1. Owned house |
| 2. Privately-rented house |
| 3. Municipally-managed house |
| 4. Rented room |
| 5. Other |
| 8) Do you receive pension benefits? Circle numbers of all answers that apply. |
| 1. National pension |
| 2. Employee pension |
| 3. Pension for public servants |
| 4. Pension fund or corporate pension |
| 5. Personal pension |
| 6. I do not receive any pension benefits |
| 9) Do you currently receive welfare benefits? |
| 1. No |
| 2. Yes |
| 3. I have applied for benefits |
| **Q14. The following questions are about your past and current jobs.** |
| 1) What is your current working status? |
| 1. I have a paid job. |
| 2. I am retired from my job. |
| 3. I have never had a job |
| 3) What type of occupation have you been engaged in for the longest in your life? |
| 1. Specialist, technician |
| 2. Manager |
| 3. Clerical worker |
| 4. Sales/service jobs |
| 5. Skills and labor |
| 6. Agriculture, forestry or fisheries |
| 7. Self-employment |
| 8. Other ( ) |
| 9. I have never had a job |
| 4) How many people worked at the company or organization where you worked the longest? |
| 1. 1 to 9 people |
| 2. 10 to 49 people |
| 3. 50 to 499 people |
| 4. 500 to 9,999 people |
| 5. 10,000 people or more |
| 6. I don’t know |
| 7. I have never had a job |

* indicates that the item is included in the Kihon Checklist.

**Supplementary Figure 1. Distribution of certified levels of needing care in the follow-up**


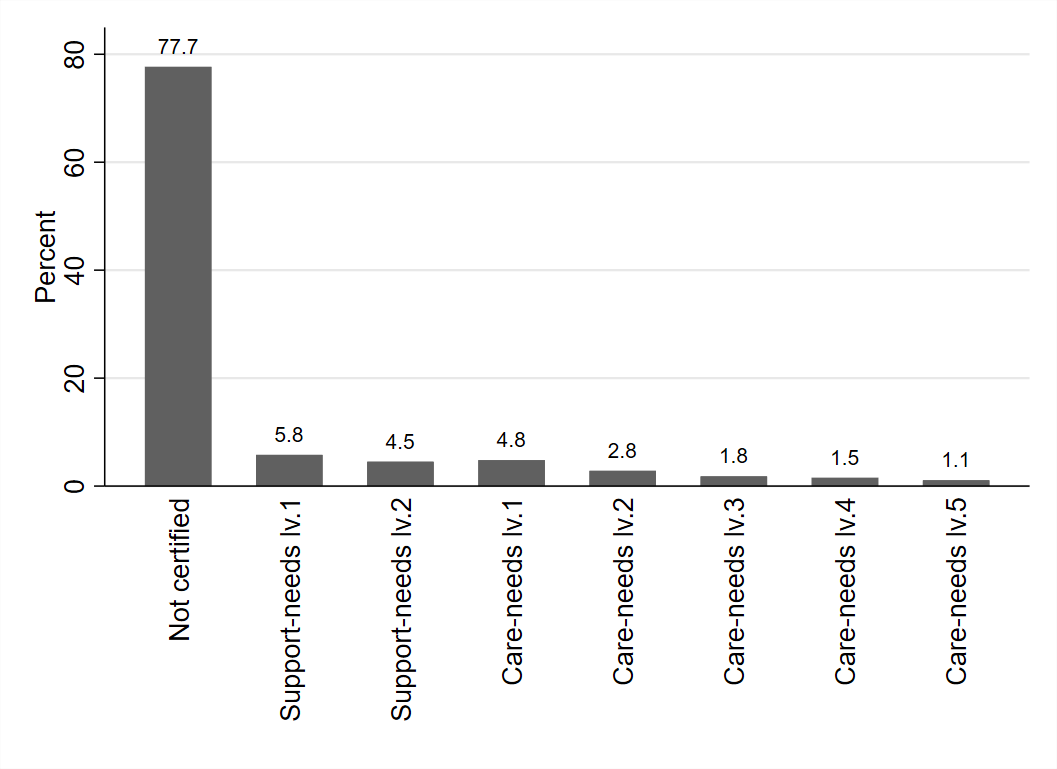


**Supplementary Table 3. Prediction performance for functional disability excluding those who were certified as needing long-term care within one year**

| Model | C statistic | Accuracy | Sensitivity | Specificity |
| --- | --- | --- | --- | --- |
| Ridge regression | 0.809 | 0.728 | 0.756 | 0.721 |
| Gradient boosting | 0.807 | 0.728 | 0.751 | 0.722 |
| Random forest | 0.792 | 0.715 | 0.742 | 0.709 |
| XGBoost | 0.792 | 0.722 | 0.73 | 0.719 |

**Supplementary Table 4. Prediction performance for the functional disability of care-needs level 2 or severer**

| Model | C statistic | Accuracy | Sensitivity | Specificity |
| --- | --- | --- | --- | --- |
| Ridge regression | 0.805 | 0.723 | 0.751 | 0.719 |
| Gradient boosting | 0.804 | 0.718 | 0.755 | 0.713 |
| Random forest | 0.783 | 0.700 | 0.740 | 0.694 |
| XGBoost | 0.783 | 0.707 | 0.733 | 0.704 |

**Supplementary Figure 2. Ten important features in the prediction models of care-needs level 2 or severer**


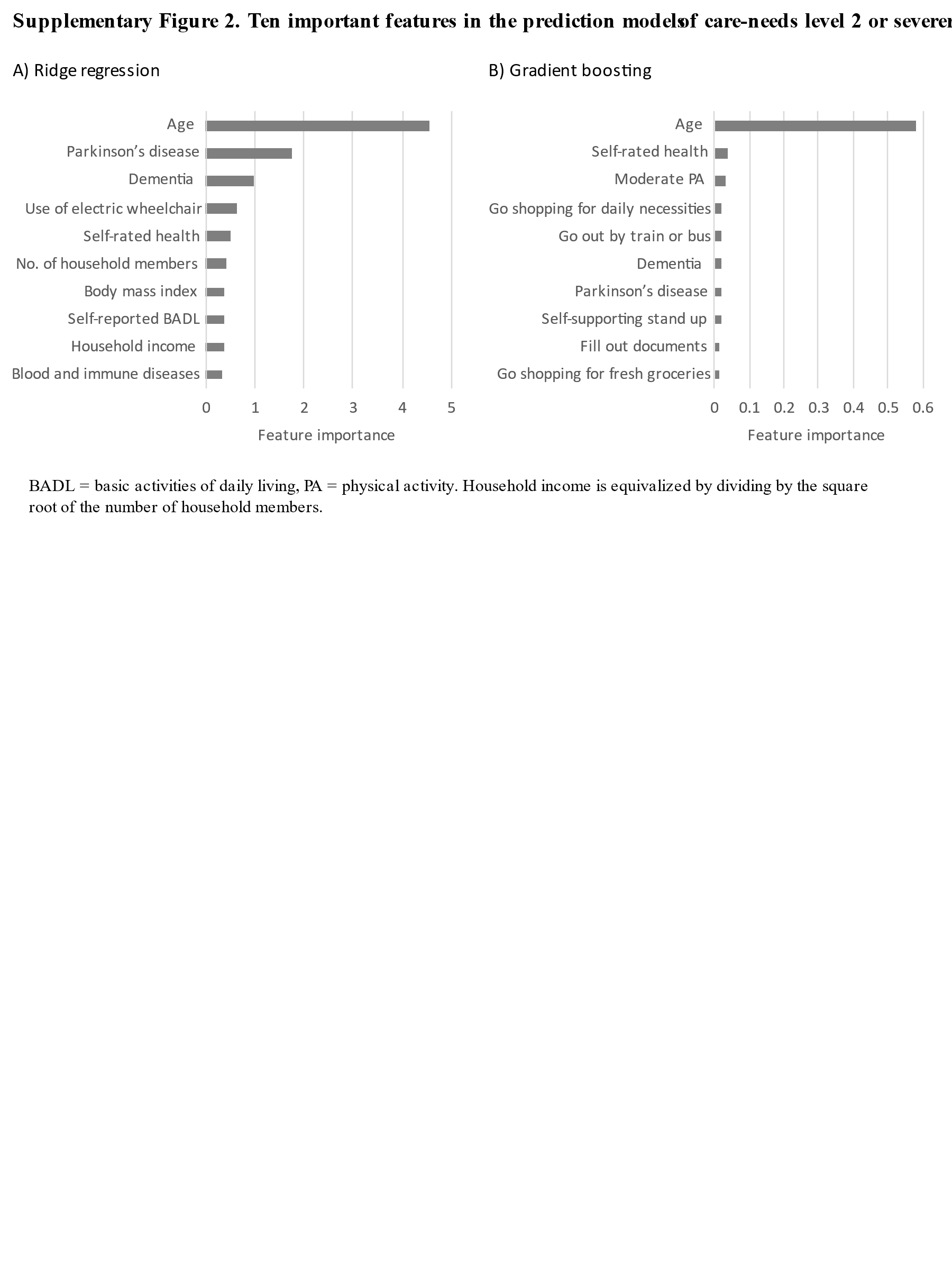


BADL = basic activities of daily living, PA = physical activity. Household income is equalized by dividing by the square root of the number of household members. Feature importance represents absolute coefficients in the ridge regression, while it represents relative values of reductions in the Gini index due to splits over a given predictor in the gradient boosting.

**Supplementary Table 5. Alternative prediction models for functional disability**

| Model | C statistic | Accuracy | Sensitivity | Specificity |
| --- | --- | --- | --- | --- |
| Cox model | 0.817 | 0.737 | 0.760 | 0.731 |
| Voting ensemble | 0.819 | 0.742 | 0.755 | 0.738 |

**Supplementary Table 6. Prediction performance of the 25-item Kihon Checklist for functional disability**

| Model | C statistic | Accuracy | Sensitivity | Specificity |
| --- | --- | --- | --- | --- |
| Ridge regression | 0.716 | 0.682 | 0.624 | 0.698 |
| Gradient boosting | 0.717 | 0.684 | 0.625 | 0.701 |
| Random forest | 0.668 | 0.656 | 0.602 | 0.672 |
| XGBoost | 0.699 | 0.666 | 0.622 | 0.679 |
